# P-1216. Pharmacokinetics of EL219 (SF001), a Novel, Next-Generation Polyene Antifungal: Results from the Phase 1 Dose-Escalation Trials in Healthy Adults and Phase 1 Open-Label Study in Adults with Moderate Renal Impairment

**DOI:** 10.1093/ofid/ofaf695.1409

**Published:** 2026-01-11

**Authors:** Taylor Sandison, Laura A Navalta, Karen Truhe, Nannette Nepomuceno, Anita F Das, Kieren A Marr

**Affiliations:** Elion Therapeutics, Encinitas, CA; Elion Therapeutics, Inc., San Diego, California; Elion Therapeutics, Encinitas, CA; Elion Therapeutics, Encinitas, CA; Das Consulting, Guerneville, CA; Elion Therapeutics, Encinitas, CA

## Abstract

**Background:**

EL219 (formerly known as SF001) is a novel, next-generation polyene antifungal designed to have long-acting, broad-spectrum, fungicidal activity and potential for reduced renal toxicity. EL219 is being developed for early antifungal therapy of suspected pulmonary mold infections, treatment of invasive aspergillosis, and treatment of cryptococcosis. The pharmacokinetics (PK) of EL219 were evaluated in Phase 1 trials conducted in healthy adults and adults with renal impairment (RI).

**Figure 1:**
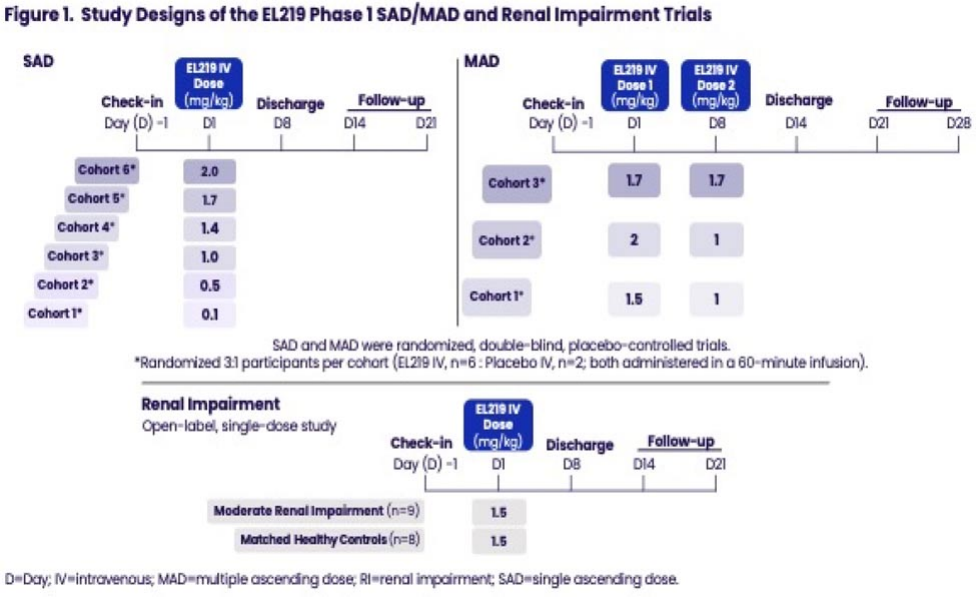
Study Designs of the EL219 Phase 1 SAD/MAD and Renal Impairment Trials

**Table 1:**
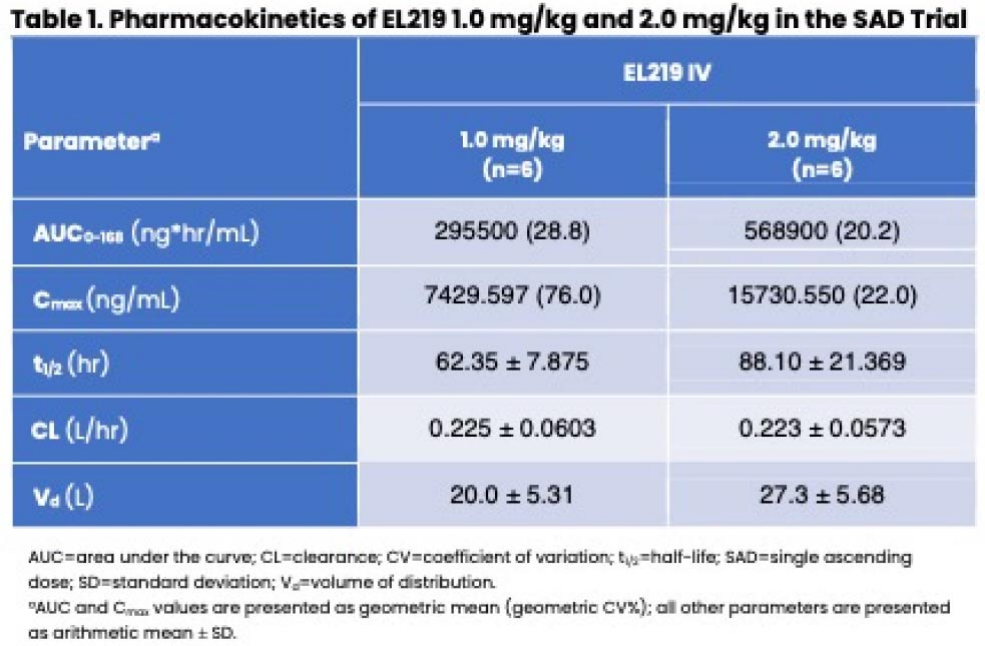
Pharmacokinetics of El219 1.0 mg/kg and 2.0 mg/kg in the SAD Trial

**Methods:**

The SAD/MAD trials evaluated the safety, tolerability, and PK of EL219 in healthy adults. The effect of RI on PK and safety of EL219 was evaluated in adults with moderate RI and matched healthy participants (controls) (Figure 1). Safety was assessed by adverse events, laboratory assessments, electrocardiograms, and vital signs (presented previously). Samples to evaluate PK were collected pre-dose and at multiple timepoints post-dose.

**Results:**

EL219 was safe and generally well tolerated in all three studies. Transient histamine-like allergic reactions, likely related to excipient (polyethylene glycol), occurred at higher doses or at higher infusion rates. Some participants showed evidence of mild, transient renal tubular abnormalities, consistent with drug aggregation, that resolved without intervention or recurrence with additional dosing. No electrolyte abnormalities, anaemia, or other safety abnormalities were observed.

Exposures as measured by AUC and C_max_ increased linearly and approximately proportionally with increasing dose in SAD, with comparable results in MAD. EL219 demonstrated a long half-life ( >60 hr following single dose administration of 1.0 and 2.0 mg/kg) (Table 1). Renal clearance was 0.2 L/hr, and urinary excretion was 20–25% as intact drug. Accumulation was ∼17% when an equivalent dose was given a week later. EL219 PK in adults with moderate RI showed a trend toward slower elimination but no significant changes compared with matched controls.

**Conclusion:**

EL219 administered in healthy adults and adults with moderate renal impairment demonstrated PK (plasma exposures, half-life, and accumulation) favorable for once-weekly dosing, and no change in dose is expected for use in adults with moderate renal impairment.

**Disclosures:**

Taylor Sandison, MD, MPH, Elion Therapeutics: Employee|Elion Therapeutics: Stocks/Bonds (Private Company) Laura A. Navalta, Bachelors of Arts, Elion Therapeutics, Inc.: Employee|Elion Therapeutics, Inc.: Stocks/Bonds (Private Company) Karen Truhe, MPH, Cidara Therapeutics: Stocks/Bonds (Private Company)|Elion Therapeutics: Advisor/Consultant|Johnson & Johnson: Stocks/Bonds (Private Company) Anita F. Das, PhD, Basilea: DSMB|Cidara: Advisor/Consultant|Elion: Advisor/Consultant|Gates MRI: DSMB|GSK: DSMB|Innoviva: DSMB|Paratek: Advisor/Consultant Kieren A. Marr, MD, MBA, Elion Therapeutics: Board Member|Elion Therapeutics: Employment|Elion Therapeutics: Stocks/Bonds (Private Company)

